# Supplementary material for: The Small RNA Universe of Capitella teleta
Source: Front Mol Biosci. 2022 Feb 25;9:802814. doi: 10.3389/fmolb.2022.802814 (PMC8915122; doi:10.3389/fmolb.2022.802814)
Supplement: Supplementary file 1 [file DataSheet1.ZIP › Supplement/known/CAPTEscaffold_114_8288.pdf]

Provisional ID	:	CAPTEscaffold_114_8288
Score total	:	5.2
Score for star read(s)	:	-1.3
Score for read counts	:	0
Score for mfe	:	1.9
Score for randfold	:	1.6
Score for cons. seed	:	3
Total read count	:	81908
Mature read count	:	81366
Loop read count	:	0
Star read count	:	542

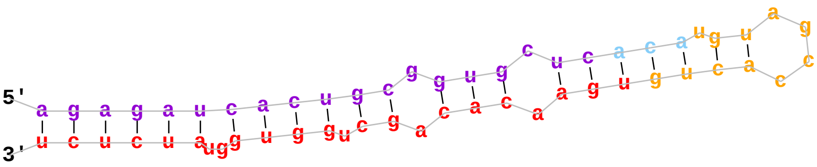

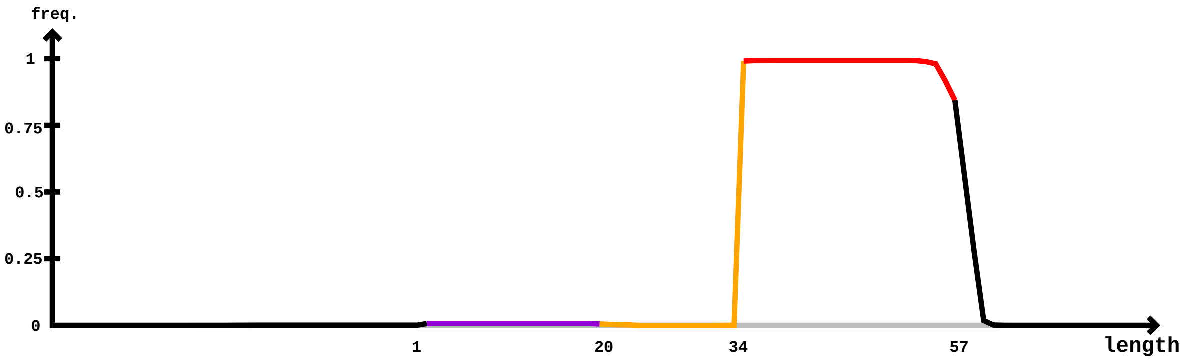


```
                                     Star                         Mature
5'- agcgcugacucggaaggagaagcgucgagcgcaggcaagagaucacugcggugcucacauguagccacugugaacacagcuggugguaucucuuuucuucgcucugaccugca  -3'   obs
    agcgcugacucggaaggagaagcgucgagcgcaggcaagagaucacugcggugcucacauguagccacugugaacacagcuggugguaucucuuuucuucgcucugaccugca        exp
    agcgcugacucggaaggagaagcgucgagcgcaggcaagagaucacugcggugcucacauguagccacugugaacacagcuggugguaucucuuuucuucgcucugaccugca        known
    .(((((..(((.....))).))))).(((((.(((.(((((((((((((.(((.(((((.((....))))))).))).)).)))).)))))))).))).)))))......... reads   mm   sample
    .......acucggaaggagaagcgucgagcgcaggca............................................................................ 3       0    seq
    ........cucggaaggagaagcgucgagcgcaggca............................................................................ 1       0    seq
    .............aaggagaagcgucgagcgcaggca............................................................................ 1       0    seq
    ..............aggagaagcgucgagcgcaggc............................................................................. 1       0    seq
    ................gagaagAgucgagcgcaggca............................................................................ 1       1    seq
    ................gagaagcgucAagcgcaggca............................................................................ 1       1    seq
    ................gagaagcgucgagcgcaggca............................................................................ 3       0    seq
    .................agaagcgucgagcAcaggca............................................................................ 1       1    seq
    .................agaagcgucgagcgcaggca............................................................................ 11      0    seq
    ..................gaagcgucgGgcgcaggca............................................................................ 1       1    seq
    ..................gaagcgucgagcgcaggca............................................................................ 8       0    seq
    ...................aagcgucgagcgcaggca............................................................................ 16      0    seq
    .....................................Ggagaucacugcggugcu.......................................................... 11      1    seq
    .....................................agagaucacugcggugcu.......................................................... 113     0    seq
    .....................................agagaucacugcggugcuc......................................................... 167     0    seq
    .....................................Ggagaucacugcggugcuc......................................................... 12      1    seq
    .....................................agagaucacugcggugcuU......................................................... 22      1    seq
    .....................................agagaucacugcggugcuUa........................................................ 1       1    seq
    .....................................agagaucacAgcggugcuca........................................................ 1       1    seq
    .....................................agagaucacugcggugcuca........................................................ 64      0    seq
    .....................................Ggagaucacugcggugcuca........................................................ 5       1    seq
    .....................................agagaucacugcggugcucac....................................................... 5       0    seq
    .....................................Ggagaucacugcggugcucaca...................................................... 9       1    seq
    .....................................agagaucacugcggugcucaca...................................................... 117     0    seq
    .....................................agagaucacugcggugcucacU...................................................... 10      1    seq
    .....................................agagaucacugcAgugcucaca...................................................... 1       1    seq
    .....................................agagaucacugUggugcucaca...................................................... 2       1    seq
    .....................................agagaucacugcggugcucaAa...................................................... 1       1    seq
    .....................................agagaucacugcgguAcucaca...................................................... 1       1    seq
    .................................................................cacugugaacacagcuggugguau........................ 1       0    seq
    ....................................................................uCugaacacagcuggugguaucucu.................... 4       1    seq
    ....................................................................uAugaacacagcuggugguaucucuu................... 1       1    seq
```

Star Mature

```
agcgcugacucggaaggagaagcgucgagcgcaggcaagagaucacugcggugcucacauguagccacugugaacacagcuggugguaucucuuuucuucgcucugaccugca
....................................................................uCugaacacagcuggugguaucucuuu..................   1     1   seq
.....................................................................Cugaacacagcuggugguaucucu....................   3     1   seq
.....................................................................Cugaacacagcuggugguaucucuu...................   3     1   seq
.....................................................................Augaacacagcuggugguaucucuuu..................   1     1   seq
.....................................................................Cugaacacagcuggugguaucucuuu..................   2     1   seq
.....................................................................Cugaacacagcuggugguaucucuuuu.................   2     1   seq
......................................................................ugaacacagcugguggua.........................   21    0   seq
......................................................................ugaacacagcuggugguU.........................   1     1   seq
......................................................................ugaacacagcugguggguau........................   309   0   seq
......................................................................ugaaAacagcuggugguau........................   1     1   seq
......................................................................ugaacacGgcuggugguau........................   1     1   seq
......................................................................ugUacacagcuggugguau........................   1     1   seq
......................................................................Agaacacagcuggugguau........................   1     1   seq
......................................................................ugaacacagAuggugguau........................   1     1   seq
......................................................................ugaGcacagcuggugguau........................   1     1   seq
......................................................................ugaacaGagcuggugguauc.......................   1     1   seq
......................................................................ugaacacagUuggugguauc.......................   3     1   seq
......................................................................ugaacacagcuAgugguauc.......................   1     1   seq
......................................................................ugaacacagcuggAgguauc.......................   1     1   seq
......................................................................ugaacaAagcuggugguauc.......................   2     1   seq
......................................................................ugaGcacagcuggugguauc.......................   1     1   seq
......................................................................Agaacacagcuggugguauc.......................   5     1   seq
......................................................................ugaacacagcugguAguauc.......................   2     1   seq
......................................................................ugaacacagcuggugguauU.......................   2     1   seq
......................................................................Ggaacacagcuggugguauc.......................   1     1   seq
......................................................................ugaacacagcuggugguauc.......................   643   0   seq
......................................................................ugaacacagcuggugguauA.......................   1     1   seq
......................................................................ugaacacagcuggugguaGcu......................   1     1   seq
......................................................................ugaacacagcAggugguaucu......................   3     1   seq
......................................................................ugaacacagcugUugguaucu......................   1     1   seq
......................................................................ugaGcacagcuggugguaucu......................   4     1   seq
......................................................................Ngaacacagcuggugguaucu......................   3     1   seq
......................................................................uAaacacagcuggugguaucu......................   8     1   seq
......................................................................ugaacacagcGggugguaucu......................   1     1   seq
......................................................................ugaacacaAcuggugguaucu......................   1     1   seq
......................................................................ugaacaGagcuggugguaucu......................   1     1   seq
......................................................................ugaacacagcugguggCaucu......................   3     1   seq
......................................................................ugUacacagcuggugguaucu......................   1     1   seq
......................................................................ugaaAacagcuggugguaucu......................   1     1   seq
......................................................................ugaacacagcuggugguaAcu......................   2     1   seq
......................................................................ugaacaUagcuggugguaucu......................   1     1   seq
......................................................................ugaacacagcuAgugguaucu......................   6     1   seq
......................................................................Ggaacacagcuggugguaucu......................   2     1   seq
......................................................................ugaacGcagcuggugguaucu......................   2     1   seq
......................................................................Agaacacagcuggugguaucu......................   26    1   seq
......................................................................ugaacacagcuggCgguaucu......................   1     1   seq
......................................................................ugaacacagcuggugguauUu......................   1     1   seq
......................................................................ugaacacagcugguggAaucu......................   4     1   seq
......................................................................ugaacacagcugguAguaucu......................   6     1   seq
......................................................................ugaacacGgcuggugguaucu......................   1     1   seq
......................................................................ugaacacagcugguCguaucu......................   1     1   seq
......................................................................ugaacacagcuggugguaucA......................   4     1   seq
......................................................................ugaacacagcuCgugguaucu......................   1     1   seq
......................................................................ugaacaAagcuggugguaucu......................   4     1   seq
......................................................................uCaacacagcuggugguaucu......................   1     1   seq
......................................................................ugaacacagcuggugguauAu......................   5     1   seq
......................................................................ugaacacaCcuggugguaucu......................   1     1   seq
......................................................................ugaacacagcuggugguaucG......................   23    1   seq
......................................................................ugaacacagcuggAgguaucu......................   3     1   seq
......................................................................ugaacacagcugguggguGucu......................   4     1   seq
......................................................................ugaacacagcuggugguaucu......................   5083  0   seq
......................................................................uUaacacagcuggugguaucu......................   1     1   seq
......................................................................ugaacacagAuggugguaucu......................   7     1   seq
......................................................................ugaacacagcugAugguaucuc.....................   1     1   seq
......................................................................ugaGcacagcuggugguaucuc.....................   14    1   seq
......................................................................ugaacacagcugguggCaucuc.....................   2     1   seq
......................................................................Ngaacacagcuggugguaucuc.....................   6     1   seq
......................................................................ugaacacagcuggugguaCcuc.....................   1     1   seq
......................................................................ugaaAacagcuggugguaucuc.....................   1     1   seq
......................................................................ugaacacagcuggugguauAuc.....................   3     1   seq
```

```
                                    Star                            Mature
agcgcugacucggaaggagaagcgucgagcgcaggcaagagaucacugcggugcucacauguagccacugugaacacagcuggugguaucucuuucuucgcucugaccugca
......................................................................ugaacacagcugCugguaucuc....................    1      1      seq
......................................................................uNaacacagcuggugguaucuc....................    1      1      seq
......................................................................ugaacacagcGggugguaucuc....................    1      1      seq
......................................................................ugaacacagcugguggAaucuc....................    5      1      seq
......................................................................ugaacacagcugguggguUucuc....................    1      1      seq
......................................................................ugaacacagcuCgugguaucuc....................    1      1      seq
......................................................................ugaacacagcuggugguaucAc....................    1      1      seq
......................................................................ugaacacagcuggugguaucuA....................    4      1      seq
......................................................................ugaacaAagcuggugguaucuc....................    1      1      seq
......................................................................ugaacacagUuggugguaucuc....................    2      1      seq
......................................................................ugGacacagcuggugguaucuc....................    2      1      seq
......................................................................ugaacacagcuggugguaucuU....................    2      1      seq
......................................................................ugaacacagcugguAguaucuc....................    3      1      seq
......................................................................Cgaacacagcuggugguaucuc....................    1      1      seq
......................................................................ugaacacagcuggugguGucuc....................    2      1      seq
......................................................................ugaacacagcuAgugguaucuc....................    11     1      seq
......................................................................ugaacacagcuggugguaucuc....................    5855   0      seq
......................................................................ugaacaUagcuggugguaucuc....................    1      1      seq
......................................................................ugaacacagcuggugguauUuc....................    1      1      seq
......................................................................Agaacacagcuggugguaucuc....................    24     1      seq
......................................................................uUaacacagcuggugguaucuc....................    1      1      seq
......................................................................ugaacacagAuggugguaucuc....................    6      1      seq
......................................................................ugaacacagcAggugguaucuc....................    3      1      seq
......................................................................uAaacacagcuggugguaucuc....................    14     1      seq
......................................................................ugaacacagcuggugguaucGcu...................    2      1      seq
......................................................................ugaacacagcCggugguaucucu...................    4      1      seq
......................................................................ugaacacagcuggugAuaucucu...................    3      1      seq
......................................................................ugGacacagcuggugguaucucu...................    2      1      seq
......................................................................ugaacacagcuggugguaucucA...................    28     1      seq
......................................................................ugaacacGgcuggugguaucucu...................    4      1      seq
......................................................................ugaacacagcuggugguaucCcu...................    2      1      seq
......................................................................ugaacacagcuggugguaucuUu...................    7      1      seq
......................................................................ugaacacagcuggugguaGcucu...................    3      1      seq
......................................................................ugaaGacagcuggugguaucucu...................    1      1      seq
......................................................................ugaacacaCcuggugguaucucu...................    1      1      seq
......................................................................Ggaacacagcuggugguaucucu...................    16     1      seq
......................................................................uAaacacagcuggugguaucucu...................    78     1      seq
......................................................................ugaacacagcuggugguaucuAu...................    14     1      seq
......................................................................ugaacacagcuggugCuaucucu...................    1      1      seq
......................................................................ugaacacagcugguggAaucucu...................    15     1      seq
......................................................................Cgaacacagcuggugguaucucu...................    9      1      seq
......................................................................Agaacacagcuggugguaucucu...................    141    1      seq
......................................................................ugaaAacagcuggugguaucucu...................    5      1      seq
......................................................................ugaacacagcuggugguaucucC...................    5      1      seq
......................................................................ugaacacagcuggugguaAcucu...................    9      1      seq
......................................................................ugaaUacagcuggugguaucucu...................    4      1      seq
......................................................................ugaacacagcGggugguaucucu...................    3      1      seq
......................................................................ugaacacagcuAgugguaucucu...................    62     1      seq
......................................................................ugaacaUagcuggugguaucucu...................    8      1      seq
......................................................................ugaacacagcAggugguaucucu...................    14     1      seq
......................................................................ugaacacagcuggugUuaucucu...................    2      1      seq
......................................................................ugaacacagcugguAguaucucu...................    33     1      seq
......................................................................ugaacacagcuCgugguaucucu...................    2      1      seq
......................................................................ugaacacagcuggugguauAucu...................    17     1      seq
......................................................................ugaacacagcuggCgguaucucu...................    4      1      seq
......................................................................ugaacacagcuggugguUucucu...................    6      1      seq
......................................................................ugaacaAagcuggugguaucucu...................    16     1      seq
......................................................................ugaacacagcuggugguauUucu...................    1      1      seq
......................................................................ugaacacagcuggAgguaucucu...................    13     1      seq
......................................................................ugaacaGagcuggugguaucucu...................    4      1      seq
......................................................................ugaacacagGuggugguaucucu...................    2      1      seq
......................................................................uCaacacagcuggugguaucucu...................    8      1      seq
......................................................................ugaGcacagcuggugguaucucu...................    19     1      seq
......................................................................ugaacacagcuUgugguaucucu...................    3      1      seq
......................................................................ugaacacagcuggugguauGucu...................    1      1      seq
......................................................................ugaacUcagcuggugguaucucu...................    5      1      seq
......................................................................ugaacacagcuggugguaucucG...................    46     1      seq
......................................................................ugaacacagcugAugguaucucu...................    5      1      seq
......................................................................ugaacacagcuggugguaucucu...................    35389  0      seq
......................................................................ugaacacagcugUugguaucucu...................    2      1      seq
```

```
                                    Star                            Mature
agcgcugacucggaaggagaagcgucgagcgcaggcaagagaucacugcggugcucacauguagccacugugaacacagcuggugguaucucuuuucuucgcucugaccugca
......................................................................ugaacGcagcuggugguaucucu....................  8     1   seq
......................................................................ugaacacagcuggugguaucuGu....................  1     1   seq
......................................................................ugaacacagcugguCguaucucu....................  1     1   seq
......................................................................ugaacacagUuggugguaucucu....................  5     1   seq
......................................................................Ngaacacagcuggugguaucucu....................  10    1   seq
......................................................................ugaacacagcugCugguaucucu....................  3     1   seq
......................................................................ugaacacagcuggugguaCcucu....................  8     1   seq
......................................................................ugaUcacagcuggugguaucucu....................  5     1   seq
......................................................................ugaacCcagcuggugguaucucu....................  1     1   seq
......................................................................ugaacacagcuggugguaucAcu....................  12    1   seq
......................................................................ugaacacagcugguggCaucucu....................  8     1   seq
......................................................................ugaacacagcuggugguGucucu....................  6     1   seq
......................................................................uUaacacagcuggugguaucucu....................  4     1   seq
......................................................................ugaacacagAuggugguaucucu....................  33    1   seq
......................................................................ugaacaAagcuggugguaucucuu...................  3     1   seq
......................................................................ugaacacagcuggugguaucuUuu...................  2     1   seq
......................................................................ugaacacagcCggugguaucucuu...................  1     1   seq
......................................................................ugaacacagcuggugAuaucucuu...................  1     1   seq
......................................................................ugaacacagcuggCgguaucucuu...................  3     1   seq
......................................................................ugaacacagcugguggCaucucuu...................  1     1   seq
......................................................................ugaacacagcugguggAaucucuu...................  4     1   seq
......................................................................ugaGcacagcuggugguaucucuu...................  14    1   seq
......................................................................ugaaAacagcuggugguaucucuu...................  1     1   seq
......................................................................ugaacacagcuAgugguaucucuu...................  27    1   seq
......................................................................ugaacacagcuggugguaucuAuu...................  3     1   seq
......................................................................uAaacacagcuggugguaucucuu...................  24    1   seq
......................................................................ugaacacagcuUgugguaucucuu...................  3     1   seq
......................................................................ugaacacGgcuggugguaucucuu...................  3     1   seq
......................................................................ugaacacagcuggugguaucucGu...................  1     1   seq
......................................................................ugaacacagcuggugguaucucNu...................  1     1   seq
......................................................................ugaacacagcuggugguaucucAu...................  5     1   seq
......................................................................ugaacacagcAggugguaucucuu...................  4     1   seq
......................................................................uCaacacagcuggugguaucucuu...................  2     1   seq
......................................................................ugaacaUagcuggugguaucucuu...................  4     1   seq
......................................................................ugaacacaUcuggugguaucucuu...................  2     1   seq
......................................................................uUaacacagcuggugguaucucuu...................  1     1   seq
......................................................................Cgaacacagcuggugguaucucuu...................  1     1   seq
......................................................................ugaacacagcugAugguaucucuu...................  3     1   seq
......................................................................ugaacacagcuggugguaucucCu...................  3     1   seq
......................................................................ugaacacagcuggugguaucucuC...................  2     1   seq
......................................................................ugaacacagcuggugguaucCcuu...................  1     1   seq
......................................................................ugaaUacagcuggugguaucucuu...................  2     1   seq
......................................................................Ngaacacagcuggugguaucucuu...................  5     1   seq
......................................................................ugaacacagcuggugguauAucuu...................  9     1   seq
......................................................................ugaacacagcuggugNuaucucuu...................  1     1   seq
......................................................................ugaacacagcuggugguaucAcuu...................  6     1   seq
......................................................................ugaacGcagcuggugguaucucuu...................  3     1   seq
......................................................................ugaacUcagcuggugguaucucuu...................  4     1   seq
......................................................................Ggaacacagcuggugguaucucuu...................  7     1   seq
......................................................................ugaacacagAuggugguaucucuu...................  11    1   seq
......................................................................ugaacacagcuggugguGucucuu...................  2     1   seq
......................................................................ugaacacagcuggugguaucucuG...................  3     1   seq
......................................................................ugaacacagcuggugguaucucuA...................  40    1   seq
......................................................................ugaacacagcuggugguaucGcuu...................  1     1   seq
......................................................................ugUacacagcuggugguaucucuu...................  2     1   seq
......................................................................ugaacacagcuCgugguaucucuu...................  1     1   seq
......................................................................ugaacacagcuggugguaucucuu...................  9892  0   seq
......................................................................ugaacacagcugguAguaucucuu...................  25    1   seq
......................................................................Agaacacagcuggugguaucucuu...................  30    1   seq
......................................................................ugaacacagcuggGgguaucucuu...................  1     1   seq
......................................................................ugGacacagcuggugguaucucuu...................  1     1   seq
......................................................................ugaacacagcuggugguauUucuu...................  1     1   seq
......................................................................ugaUcacagcuggugguaucucuu...................  1     1   seq
......................................................................ugaacacagcuggAgguaucucuu...................  8     1   seq
......................................................................ugaacacagcuggugguaAcucuu...................  5     1   seq
......................................................................ugaacacagcAggugguaucucuuu..................  11    1   seq
......................................................................ugaacacagcuggugguaucucuAu..................  17    1   seq
......................................................................ugaacacagcuggugguauUucuuu..................  3     1   seq
......................................................................ugaaGacagcuggugguaucucuuu..................  1     1   seq
......................................................................ugaacacagcuggugguaucucuGu..................  3     1   seq
```

```
                                    Star                            Mature
agcgcugacucggaaggagaagcgucgagcgcaggcaagagaucacugcggugcucacauguagccacugugaacacagcuggugguaucucuuuucuucgcucugaccugca
......................................................................ugaacacagcugCugguaucucuuu..................   3      1   seq
......................................................................ugaacacagcuggugguaucAcuuu..................   9      1   seq
......................................................................ugaacacagcuggugguaCcucuuu..................   1      1   seq
......................................................................ugaacacagAuggugguaucucuuu..................   13     1   seq
......................................................................ugaacacagcuggugguaAcucuuu..................   4      1   seq
......................................................................ugaacacagcuggugguaucucuCu..................   2      1   seq
......................................................................Ngaacacagcuggugguaucucuuu..................   5      1   seq
......................................................................ugaacacagcuAgugguaucucuuu..................   50     1   seq
......................................................................ugaacacagcuggugguUucucuuu..................   1      1   seq
......................................................................ugaacUcagcuggugguaucucuuu..................   2      1   seq
......................................................................ugaacacaUcuggugguaucucuuu..................   3      1   seq
......................................................................ugaacacagcugguggCaucucuuu..................   2      1   seq
......................................................................ugaacacagUuggugguaucucuuu..................   2      1   seq
......................................................................ugaacacagcCggugguaucucuuu..................   1      1   seq
......................................................................ugaacacagcuCgugguaucucuuu..................   2      1   seq
......................................................................ugaacacagcuggugguauAucuuu..................   14     1   seq
......................................................................ugaacacagcuggugAuaucucuuu..................   1      1   seq
......................................................................ugaacacagcuggCgguaucucuuu..................   3      1   seq
......................................................................ugaacaUagcuggugguaucucuuu..................   2      1   seq
......................................................................Ggaacacagcuggugguaucucuuu..................   9      1   seq
......................................................................ugaacacagcuggugguaucuAuuu..................   6      1   seq
......................................................................ugaacacagcuggugguaucucuuG..................   5      1   seq
......................................................................ugaaAacagcuggugguaucucuuu..................   3      1   seq
......................................................................ugaacacGgcuggugguaucucuuu..................   2      1   seq
......................................................................ugaGcacagcuggugguaucucuuu..................   15     1   seq
......................................................................ugaacacagcuggugguaucucuuC..................   7      1   seq
......................................................................Agaacacagcuggugguaucucuuu..................   90     1   seq
......................................................................ugaacaAagcuggugguaucucuuu..................   7      1   seq
......................................................................ugGacacagcuggugguaucucuuu..................   3      1   seq
......................................................................ugaacacagcuggugguaucucuuA..................   40     1   seq
......................................................................ugaacacagcuggugguaucCcuuu..................   2      1   seq
......................................................................ugaacacagcuggGgguaucucuuu..................   1      1   seq
......................................................................ugaacacagcugguCguaucucuuu..................   3      1   seq
......................................................................ugaacacagcGggugguaucucuuu..................   1      1   seq
......................................................................ugaacacagcuggugguaucGcuuu..................   1      1   seq
......................................................................ugaacacagcugguAguaucucuuu..................   32     1   seq
......................................................................uCaacacagcuggugguaucucuuu..................   1      1   seq
......................................................................ugaacacagcuggAgguaucucuuu..................   5      1   seq
......................................................................ugaacacagcuggugguaucucAuu..................   4      1   seq
......................................................................Cgaacacagcuggugguaucucuuu..................   5      1   seq
......................................................................ugaacacagcugguggAaucucuuu..................   4      1   seq
......................................................................ugaacacagcuggugguGucucuuu..................   9      1   seq
......................................................................ugaacaGagcuggugguaucucuuu..................   2      1   seq
......................................................................ugaacacagcuggugUuaucucuuu..................   2      1   seq
......................................................................ugaacacagcuggugCuaucucuuu..................   2      1   seq
......................................................................ugaacacagcuggugguaucucCuu..................   12     1   seq
......................................................................ugaaUacagcuggugguaucucuuu..................   1      1   seq
......................................................................ugaacacagcuggugguaucuUuuu..................   2      1   seq
......................................................................uAaacacagcuggugguaucucuuu..................   63     1   seq
......................................................................ugaacacagGuggugguaucucuuu..................   1      1   seq
......................................................................ugUacacagcuggugguaucucuuu..................   3      1   seq
......................................................................ugaacGcagcuggugguaucucuuu..................   3      1   seq
......................................................................ugaacacagcugguggGaucucuuu..................   2      1   seq
......................................................................ugaacacagcugguUguaucucuuu..................   2      1   seq
......................................................................ugaacacagcuUgugguaucucuuu..................   1      1   seq
......................................................................ugaacacagcugUugguaucucuuu..................   1      1   seq
......................................................................ugaacacaAcuggugguaucucuuu..................   2      1   seq
......................................................................ugaacacagcugAugguaucucuuu..................   1      1   seq
......................................................................ugaacacagcuggugguaucucuuu..................   20731  0   seq
......................................................................uUaacacagcuggugguaucucuuu..................   3      1   seq
......................................................................ugaacacagcuggugguaucucGuu..................   1      1   seq
......................................................................ugaacacagcuggugguaGcucuuu..................   3      1   seq
......................................................................ugaacacagcuggugguaucucuuuu.................   1167   0   seq
......................................................................ugaacacagcuAgugguaucucuuuu.................   4      1   seq
......................................................................ugaacacagcuggugguaAcucuuuu.................   1      1   seq
......................................................................ugaacacagcuggugguaCcucuuuu.................   1      1   seq
......................................................................ugaacacagcuggugguaucucuuuC.................   3      1   seq
......................................................................ugaacacagcugguCguaucucuuuu.................   2      1   seq
......................................................................ugaacacagcuggugguaucucuuCu.................   4      1   seq
......................................................................Agaacacagcuggugguaucucuuuu.................   6      1   seq
```

```
                                     Star                             Mature
agcgcugacucggaaggagaagcgucgagcgcaggcaagagaucacugcggugcucacauguagccacugugaacacagcuggugguaucucuuuucuucgcucugaccugca
......................................................................ugaacacaAcuggugguaucucuuuu.................   1     1     seq
......................................................................ugaacacagcuggugguauAucuuuu.................   1     1     seq
......................................................................ugaacacagcuggugguaucucuuuA.................   157   1     seq
......................................................................ugaacacagcAggugguaucucuuuu.................   1     1     seq
......................................................................ugaacacagcuggugAuaucucuuuu.................   1     1     seq
......................................................................ugGacacagcuggugguaucucuuuu.................   1     1     seq
......................................................................ugaacacagcuggugguaucucuuAu.................   5     1     seq
......................................................................ugaacacagcugguAguaucucuuuu.................   3     1     seq
......................................................................ugaacacagcuggugguaucucuuuG.................   1     1     seq
......................................................................ugaacacagcuggugguGucucuuuu.................   1     1     seq
......................................................................ugaacacagAuggugguaucucuuuu.................   1     1     seq
......................................................................ugaacacaCcuggugguaucucuuuu.................   1     1     seq
......................................................................ugaaUacagcuggugguaucucuuuu.................   1     1     seq
......................................................................ugaacacagcuggugguaucucuuuuU................   83    1     seq
......................................................................ugaacacagcuggugguaucucuuuuA................   1     1     seq
......................................................................ugaacacagcuggugguaucucuuuuUu...............   8     1     seq
......................................................................ugaacacagcuggugguaucucuuuuAu...............   2     1     seq
......................................................................ugaacacagcuggugguaucucuuuucu...............   1     0     seq
......................................................................ugaacacagcuggugguaucucuuuuUuu..............   2     1     seq
.......................................................................gaacacagcuggugguau........................   2     0     seq
.......................................................................gaacacagcuggugguaucu......................   6     0     seq
.......................................................................gaacacagcuggugguaucG......................   1     1     seq
.......................................................................gaacacagcuggugguaucuc.....................   6     0     seq
.......................................................................gaacacagcuggAgguaucucu....................   1     1     seq
.......................................................................gaacacagcuggugguaucucu....................   52    0     seq
.......................................................................Uaacacagcuggugguaucucu....................   3     1     seq
.......................................................................gaacacagcuggugguaucucuu...................   20    0     seq
.......................................................................gaacacagcuggugguaucucuuu..................   31    0     seq
.......................................................................Uaacacagcuggugguaucucuuu..................   1     1     seq
.......................................................................gaacacagcuggugguaucucuuuu.................   3     0     seq
........................................................................aacacagcuggugguaucucu....................   1     0     seq
........................................................................aacacagcuggugguaucucuuu..................   3     0     seq
.........................................................................acacagcuggugguaucu......................   2     0     seq
.........................................................................acacagcuggugguaucuc.....................   6     0     seq
.........................................................................acacagcuggugguaucucu....................   2     0     seq
.........................................................................acacagcuggugguaucucuuu..................   2     0     seq
..........................................................................cacagcuggugguaucucuu...................   3     0     seq
............................................................................cagcuggugguaucucuu...................   2     0     seq
............................................................................cagcuggugguaucucuuu..................   1     0     seq
```
